# Temporal contiguity determines overshadowing and potentiation of human Action-Outcome performance

**DOI:** 10.3758/s13423-022-02155-4

**Published:** 2022-08-11

**Authors:** José A. Alcalá, Richard D. Kirkden, Jess Bray, José Prados, Gonzalo P. Urcelay

**Affiliations:** 1 School of Psychology, University of Nottingham, University Park, Nottingham, NG7 2RD UK; 2 Department of Neuroscience, Psychology and Behaviour, University of Leicester, Leicester, UK; 3 School of Psychology, University of Derby, Derby, UK

**Keywords:** Cue competition, Overshadowing, Potentiation, Temporal contiguity, Action-outcome

## Abstract

Three experiments (*n* = 81, *n* = 81, *n* = 82, respectively) explored how temporal contiguity influences Action-Outcome learning, assessing whether an intervening signal competed, facilitated, or had no effect on performance and causal attribution in undergraduate participants. Across experiments, we observed competition and facilitation as a function of the temporal contiguity between Action and Outcome. When there was a strong temporal relationship between Action and Outcome, the signal competed with the action, hindering instrumental performance but not causal attribution (Experiments 1 and 3). However, with weak temporal contiguity, the same signal facilitated both instrumental performance and causal attribution (Experiments 1 and 2). Finally, the physical intensity of the signal determined the magnitude of competition. As anticipated by associative learning models, a more salient signal attenuated to a greater extent instrumental performance (Experiment 3). These results are discussed by reference to a recent adaptation of the configural theory of learning.

**Supplementary Information:**

The online version contains supplementary material available at 10.3758/s13423-022-02155-4.

## Introduction

When multiple sources of information (e.g., a meal’s color and flavor) predict an outcome (e.g., poisoning), organisms tend to select one source at the expense of the other. Understanding how organisms learn about multiple antecedents is one of the foundational aims of theories of learning and cognition. This is complex because there are several types of antecedents that can interact: two potential cues predicting an outcome, multiple actions (e.g., lever and chain press), or a combination of an action and a cue. The current study explored this latter interaction.

The most common outcome of the interaction is a decrease in the extent to which a target event (cue or action) controls behavior compared to when training occurs in the absence of redundant events (hereafter, competition). This happens when the target event (X) is trained simultaneously in the presence of a concurrent event (A), (overshadowing – Pavlov, 1927), or when X is paired with an already pre-trained event (blocking – Kamin, 1969). However, in some circumstances, the opposite results are observed: learning about X is potentiated by the presence of additional events (Urcelay & Miller, 2009). This discrepancy suggests that the interaction might vary in a continuum, from competition to facilitation, depending on particular environmental conditions. Notably, this pattern is observed across species and learning domains (Urcelay, 2017), suggesting that the learning mechanisms underlying these interactions are domain general (Heyes, 2019). Associative learning theories have provided an extraordinarily fruitful framework to study these interactions. However, most (if not all) associative models have primarily focused on competition, making no predictions about when facilitation should occur (Shanks, 2010; Wasserman & Miller, 1997). Although accounts of facilitation have been proposed (e.g., Durlach & Rescorla, 1980), no single model accounts for competition and facilitation in the same framework.

For example, two prominent models of associative learning explain overshadowing by different mechanisms. The elemental model by Rescorla and Wagner (1972) states that when learning about X takes place in the presence of cue A, each event will acquire a proportion of the total associative strength that the outcome supports. Hence X will acquire roughly half of the associative strength compared to when trained without A. Conversely, Pearce's configural model (1987) assumes that stimuli are processed as a configural unit (AX), and this unit enters into association with the outcome. Hence, overshadowing is accounted for by a generalization decrement from the compound (AX) to its elements tested in isolation (X), the magnitude of the decrement being a function of the similarity between the elements (see *Discussion*). One model assumes that learning is always elemental, while the other model assumes that learning is always configural. However, several findings suggest that both elemental and configural learning can develop, depending on different factors (see Melchers et al., 2008).

Indeed, flexible encoding has been proposed as a determinant of competition and facilitation. While elemental processing promotes competition, configural processing seems to lead to facilitation or (at least) attenuates competition (Urcelay & Miller, 2009; Williams et al., 1994). However, a burgeoning question is what variables determine the outcome of the interaction. Urcelay (2017) identified temporal and spatial proximity between events as a relevant factor: across different species and learning domains, contiguity determines whether the interaction between multiple antecedents is competitive or synergistic (Batsell et al., 2012; Cunha et al., 2015; Herrera et al., 2022; Schachtman et al., 1987). Despite the consistency at an empirical level, the aforementioned models are somewhat silent on what effect manipulations of contiguity should have on learning about multiple antecedents.

Building on the challenge to integrate contiguity at a theoretical level, Herrera et al. (2022) proposed an amendment to Pearce’s configural theory (1987). Briefly, we proposed that strong contiguity between events yields competition, presumably by promoting elemental processing of the information. However, weakening contiguity should promote configural processing of the information, resulting in more transfer from the AX compound to the test event X (see *Discussion*). Therefore, competition or facilitation are predicted based on temporal contiguity, consistent with a flexible encoding approach (Melchers et al., 2008).

We assessed the rationale proposed by Herrera et al. (2022), testing whether temporal contiguity plays a critical role in Action-Outcome learning. Action-Outcome learning was experienced along with (or not) an intervening event (i.e., signal). Given that the signal also predicts the outcome, standard associative theories predict that this signal should compete with the action (e.g., Rescorla & Wagner, 1972). However, we anticipated that the signal would only compete with the action with strong Action-Outcome temporal contiguity, instead the signal would facilitate Action-Outcome learning with weak contiguity – this was tested in Experiment 1. Experiments 2 and 3 further investigated conditions that presumably promote elemental or configural encoding, resulting in competition and facilitation, respectively.

## Experiment 1

We manipulated the Action-Outcome temporal contiguity and the presence (or absence) of an intervening signal using a free-operant procedure. A brief signal (0.5 s) that did not fill the entire delay was used (e.g., Pearce & Hall, 1978), ensuring that the signal did not serve to entirely bridge the delay between Action-Outcome by providing sensory feedback (cf., Shanks, 1989). We expected that the signal would compete with acquisition of Action-Outcome learning with strong Action-Outcome temporal contiguity (2-s delay), but the same signal would potentiate Action-Outcome learning with weak contiguity (6-s delay).^1^

### Method

#### Participants

Eighty-one undergraduate students (eight men), with an average age of 19.56 years (range 18–30) participated in the experiment and were compensated with course credit. The experiment was run in two replications. In Replication 1 (*n* = 30) participants were recruited at the University of Leicester (UK) and the experiment took place in the laboratory. In Replication 2, which was run remotely (*n* = 51), participants were from the University of Jaén (Spain) and participated online in the experiment. The differences in the recruitment of the participants were caused by COVID-19 and the closing of the laboratory. Participants were not instructed about the specific goal of the task and had no previous experience with it. The experiment was approved by the Ethics Committee at the University of Leicester, application reference number 20955.

No specific power analysis to calculate the sample size was conducted. However, given that part of the sample was tested online, we decided to use a large sample of participants. Classic and current studies using free-operant procedures usually include samples of between 10 and 60 participants (e.g., Greville & Buehner, 2010; Pérez & Soto, 2020; Reed, 1996, 1999; Shanks et al., 1989), hence, prior to data collection, we decided to use a larger sample compared to previous studies using similar procedures. Sensitivity analyses using the software G*power revealed that with a sample of 81 participants, the smallest effect size that could be detected for the critical simple effect of Signal with a power of .90 and an alpha criterion of .05 was *F*(1,80) = 3.96, η^*2*^_*p*_ = .03. Note that this effect is smaller than the actual effect size observed in the experiments reported here, suggesting strong sensitivity in our sample to competition and facilitation effects.

#### Design

A 2 (Replication: 1 vs. 2) × 2 (Contingency: Fixed Ratio 1 vs. Partial Reinforcement) × 2 (Delay: 2 s vs. 6 s) × 2 (Signal: Signal vs. No-Signal) mixed design was used. Replication was included as a between-subjects variable in order to control for differences between laboratory and virtual data collection. All other factors were manipulated within-subjects. Contingency had two different levels determining the relationship between action and the outcome: Fixed Ratio 1 (FR1; each response triggered an outcome) and Partial Reinforcement (PR; each response triggered the outcome with a variable probability of 2/3). The outcome was delayed 2 s (strong contiguity) and 6 s (weak contiguity). In the No-Signal condition there was no signal between the action and outcome (control condition), whereas in the Signal condition a 0.5-s signal was presented 1.5 s after each reinforced action (experimental condition). Two levels of contingency were used to increase sampling (we collected two measures in each level of Delay and Signal) while making the different conditions distinctive to avoid the same pattern of behavior across conditions.

#### Apparatus

The task used in this study was programmed in PsychoPy2 (Peirce et al., 2019). In Replication 1, participants ran the experiment in an individual cubicle at the University of Leicester. Each cubicle had a 19-in. AG Neovo F-419 LCD screen attached to a Hewlett-Packard Compaq Elite 8300 PC desktop computer, running Windows 10. In Replication 2, participants ran the task in their homes. Each participant used their own computer with the aforementioned version of PsychoPy.

#### Procedure

In Replication 1, the participants were tested in individual rooms. In Replication 2, the experimenter was connected via a videoconference with a group of 3–4 participants. Participants were encouraged to be alone in a quiet room, with the computer on a desk, their phone in a different room, and to avoid any type of music or noise in the background. At the beginning of the videoconference, the experimenter emailed the program to the participants and guided them to install and open PsychoPy.

After reading and signing the consent form, participants were presented with visual instructions for the task. The instructions read (in English in Replication 1, Spanish in Replication 2):

The year is 3020 and your city is under attack from an alien invasion of a new ‘mushroom’ species.

Your task is to shoot at the aliens by pressing the SPACE BAR to protect your city. You can press as often or as little as you please.

The sky may flash at times, indicating that your weapon or that of one of your fellow comrades is shooting.

Afterwards, you must judge from this to what extent the explosions are due to your shooting and will be asked to rate this from 0-100 after each condition.

If you have any questions please ask the researcher, if not please press RETURN to begin.

In the first replication, we included an additional sentence stating: *“However, it is in your best interest to conserve your ammo and not fire constantly.”* For the second replication, and for the rest of the experiments, we eliminated this sentence, to minimize biasing the participant’s response rate. After reading the instructions, participants in both replications were asked whether they fully understood them, and any further questions were answered. In the first replication, participants were alone in the cubicle while in the second replication microphones and cameras for experimenter and participants were switched off (although the muted virtual conference remained open for subsequent data extraction).

**Fig. 1:**
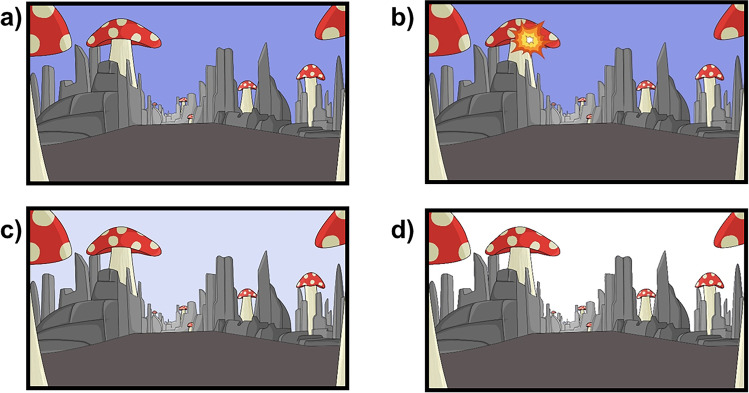
Snapshots of the different images used in the game. Panel (**a**) displays the default scenario that participants experienced in the absence of any other events. Panel (**b**) shows aSnapshot of the delivery of the outcome (0.1-s length). Panel (**c**) represents the grey sky used as Low Signal in Experiments 1–3 (0.5-s length). Panel (**d**) characterizes the white sky used as High Signal in Experiment 3 (0.5-s length)

**Fig. 2:**
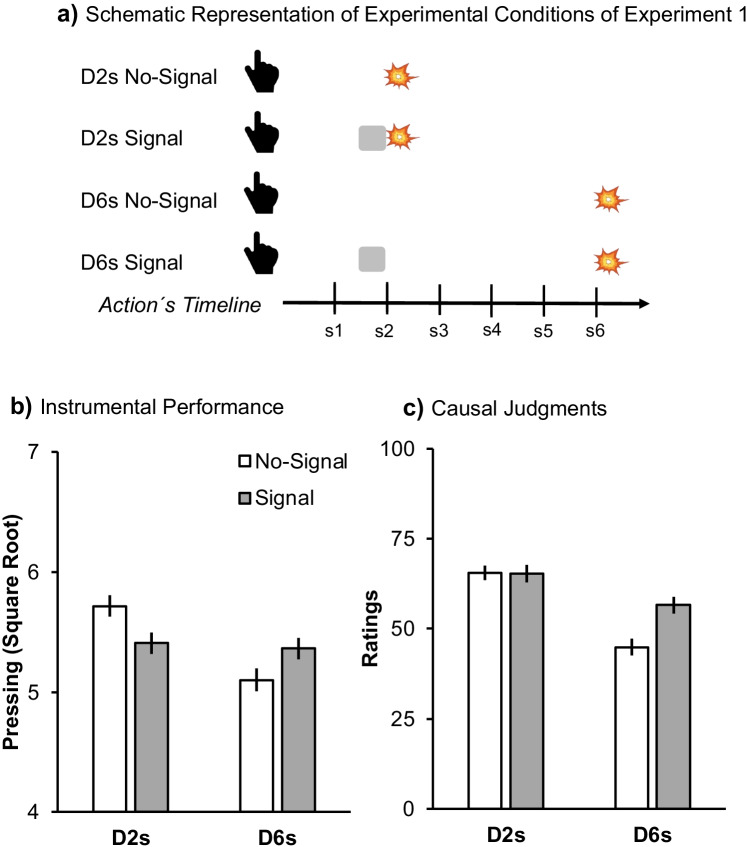
Design and results of Experiment 1. Panel (**a**) depicts the schematic timeline of each experimental condition for Experiment 1. D2s refers to the condition in which the outcome was delayed 2 s and D6s refers to 6-s delay. The hand symbolizes the action starting the timeline, the grey square represents the signal, and the explosion the outcome. Panel (**b**) represents the mean square root of the number of presses; Panel (**c**) depicts the mean of causal judgments. Error bars are SEM applying the within-subjects correction suggested by O’Brien and Cousineau (2014)

The task followed the basic structure of other free-operant procedures. During each condition, participants were exposed to the scenario shown in Fig. 1a. When playing the computer game, participants’ presses of the space bar (Action) triggered an explosion that appeared for 0.1 s (Outcome; see Fig. 1b) on one of the mushrooms. There was a debounce time after each response (0.5 s), in which further responses were not registered and did not set up any outcomes. Participants experienced each experimental condition for 2 min, followed by a test question about this particular condition. There were eight experimental conditions stemming from the 2 × 2 × 2 within-subject design: 2 (Contingency: FR1 vs. Partial) × 2 (Delay: 2 s vs. 6 s) × 2 (Signal: Signal vs. No-Signal). All participants experienced each condition once in a random order (Fig. 2a depicts a schematic representation of the relevant experimental conditions).

In the probabilistic condition each action was followed by the outcome according to a variable probability of 2/3. The two levels of the factor Delay referred to the temporal interval between Action and Outcome. The Outcome was delayed for 2 s or 6 s after the Action. Finally, the factor Signal had two different levels. In the No-Signal conditions, there was no signal presented between Action and Outcome; thus, the sky remained unchanged during the delay (Fig. 1a). However, in the Signal conditions the sky changed color to grey (i.e., signal) 1.5 s after the action for 0.5 s (Fig. 1c).

In total, participants completed each permutation of the factorial design once, in random order. At the end of each condition, participants were asked to judge if they thought their action caused the outcome. Here, participants were presented with the following instructions:

*On the scale use the mouse to indicate to what extent pressing the SPACE BAR (firing your weapon) caused the explosions. The scale rates from 0 to 100.*

*0 = pressing the SPACE BAR had no effect on explosions appearing*

*100 = pressing the SPACE BAR always caused the explosions*

Participants used the mouse to move a slider along a 0–100 scale, and a number on the screen displayed the currently selected value. Participants pressed “continue” to record their judgment, after which the next condition of the experiment began automatically. It took approximately 20 min to complete the experiment.

In the first replication, participants were debriefed after finishing the experiment. In the second replication, the experimenter guided the participant to find the data file in their computer and send it to the experimenter. After receiving the data file, the experimenter debriefed participants and emailed them granting course credit.

### Data analysis

All subjects were included in the analyses and data were analyzed once the final sample of 81 participants had been reached. For the instrumental performance data, the distribution across participants was skewed. Therefore, we transformed for each participant the total number of presses in each condition of the experiment by calculating the square root of space bar presses (e.g., Greville & Buehner, 2010). For the causal judgments, we recorded the ratings after each condition

Analyses of variance (ANOVAs) were used to assess separately the instrumental performance and the causal judgments in each condition stemming from the factorial design described above. Across experiments the Contingency factor did not interact with the critical Delay by Signal interaction (Experiment 1) or the main effect of Signal (Experiments 2 and 3), except for a three-way interaction (Delay x Signal x Contingency) for the causal judgments in Experiment 1. A detailed analysis of this interaction is provided in the Online Supplementary Material (OSM), but for the rest of the *Results* section the effects of Contingency are not reported (although Contingency was included in the global analyses).

Hence, we focused on the expected Delay by Signal interaction. Critically, planned comparisons were used to evaluate the experimental (Signal) and control (No-Signal) conditions for each level of Delay. Descriptive data for these comparisons are available in Table 1. The rejection criterion was set at .05 for all statistical tests. Partial eta-squared measures were reported as effect sizes and their 95% confidence intervals (CIs) were reported using Nelson’s (2016) software. The average experienced contingency in the PR conditions was 0.66.

**Table 1:** Descriptive statistics in each condition

		Instrumental (square root)		Instrumental (raw data)		Causal judgments	
Exp.	Conditions	Mean	SD	Mean	SD	Mean	SD
1	D2 No-Signal	5.72	1.88	36.73	21.92	65.57	24.66
	D2 Signal	5.41	1.71	32.98	20.90	65.33	25.04
	D6 No-Signal	5.10	1.71	29.51	18.32	44.92	27.45
	D6 Signal	5.36	1.76	32.23	20.91	56.62	27.62
2	D6 No-Signal	5.12	2.01	31.10	22.45	30.98	25.95
	D6 Beginning	5.74	1.68	37.06	20.03	48.06	26.34
	D6 End	5.01	1.90	29.59	22.35	36.66	23.10
3	D2 No-Signal	5.95	1.83	39.79	26.24	66.10	20.50
	D2 Low-Signal	5.49	1.81	37.38	21.95	68.51	22.95
	D2 High-Signal	5.79	1.76	34.20	23.10	65.05	22.51

*Note.* Mean and standard deviation for each experimental condition, collapsing data across contingencies. Instrumental performance (number of presses) and Casual attribution (ratings) were presented. Instrumental performance is reported for the square root transformed data and the untransformed data

### Results

#### Instrumental performance

Figure 2b suggests opposite effects of the signal as a function of outcome delay. With a 2-s Action-Outcome delay the signal reduced performance compared to the control condition without signal. However, the signal increased performance with a 6-s Action-Outcome delay. A 2 (Replication: 1 vs. 2) × 2 (Contingency: FR1 vs. Partial) × 2 (Delay: 2 s vs. 6 s) × 2 (Signal: Signal vs. No-Signal) mixed-design ANOVA, revealed the critical Delay x Signal interaction, *F*(1,79) = 14.80, *p* < .001, η^*2*^_*p*_ = .16, 95% CI [.04, .30], not modulated by Replication, *F*(1,79) < 1^2^. Follow-up analyses revealed in the 2-s conditions a significant effect of Signal *F*(1,80) = 6.12, *p* = .015, η^*2*^_*p*_ = .07, [.00, .20]. In the 6-s conditions, the effect of Signal was also significant, *F*(1,80) = 4.82, *p* = .031, η^*2*^_*p*_ = .06, [.00, .18], but in the opposite direction.

#### Causal judgments

Figure 2c suggests a similar pattern in causal attribution when the outcome was delayed by 6 s, indicating facilitation. However, with a 2-s delay the signal did not result in overshadowing. The same mixed-design ANOVA indicated again the critical Delay × Signal interaction, *F*(1,79) = 6.96, *p* = .010, η^*2*^_*p*_ = .08, 95% CI [.00, .20]. Subsequent analyses revealed that in the 2-s conditions, the effect of Signal was not significant, *F*(1,80) = 0.07, *p* = .936; but it was in the 6-s conditions, *F*(1,80) = 16.50, *p* <.001, η^*2*^_*p*_ = .17, [.05, .31].

Competition and facilitation were determined by the temporal contiguity between Action-Outcome. With strong contiguity, the signal overshadowed instrumental performance, but had no effect on causal attribution. However, the same signal potentiated both performance and causal attribution with weak contiguity.

## Experiment 2

To our knowledge, data from Experiment 1 are the first evidence of opposite effects of an intervening signal as a function of contiguity in human Action-Outcome learning (Schachtman et al., 1987, in pigeons). However, in Experiment 1 Signal-Outcome contiguity was different in each level of delay. It is possible that competition only occurred because of the strong Signal-Outcome (instead of Action-Outcome) contiguity in the 2-s delay conditions. Indeed, a signal placed at the end of delay decreased instrumental performance in rodents, but facilitated it when placed at the beginning (Williams, 1999; cf., Reed, 1996, in humans).

**Fig. 3:**
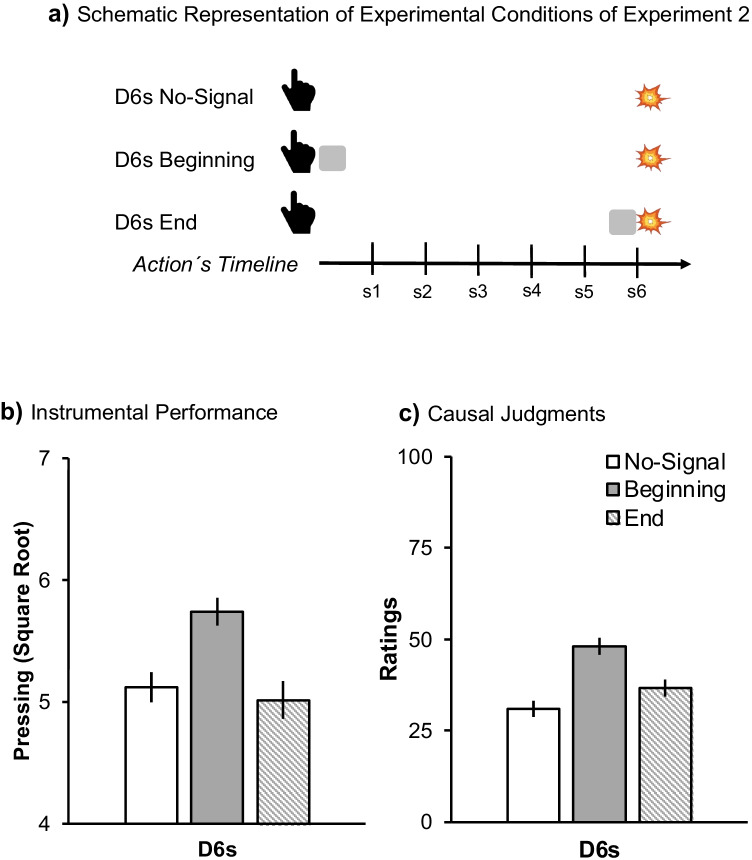
Design and results of Experiment 2. Panel (**a**) depicts the schematic timeline of each experimental condition for Experiment 2. D6s means that the outcome was delayed for 6 s. The hand symbolizes the action starting the timeline, the grey square represents the signal and the explosion the outcome. Panel (**b**) represents the mean square root of the number of presses. Panel (**c**) depicts the mean of causal judgments. Error bars are SEM applying the within-subjects correction suggested by O’Brien and Cousineau (2014)

To assess this, in Experiment 2 all conditions used weak contiguity, and the signal was presented either at the beginning or at the end of the delay (Fig. 3a). Placing the signal at the end of the delay mimicked the conditions of strong Signal-Outcome contiguity of Experiment 1. If competition was driven by Signal-Outcome contiguity rather than Action-Outcome contiguity (as hypothesized), we should observe competition (D6s End). However, given that we programmed weak Action-Outcome contiguity (promoting facilitation), these conditions might counteract each other, resulting in no interaction. Finally, moving the signal closer to the action (D6s Beginning) should increase the likelihood that Action-Signal are configured as a unit, and this should potentiate Action-Outcome learning.

### Method

#### Participants

An initial sample of 81 participants was recruited for the study. However, because of a technical issue, the causal ratings of 11 participants were not recorded. We replaced these participants with 11 new participants. Hence, in total we collected data from 92 participants but for data analyses we only considered participants with all data recorded. The final sample was composed of 81 (eight males) undergraduates from the University of Leicester with an average age of 19.48 years (range 18–40). Participants were recruited through the SONA system in exchange for course credit.

#### Procedure and design

The task was hosted on the online recruitment platform Pavlovia and programmed in PsychoPy2. The procedure was similar to the online version of Experiment 1 (Replication 2), except that participants were not connected in a virtual meeting with the experimenter. All participants experienced six different experimental conditions (see Fig. 3a) stemming from the 2 × 3 within-subject factorial design: 2 (Contingency: FR1 vs. Partial) × 3 (Signal: No-Signal, Beginning, End). Contingency levels were determined as in Experiment 1. In the condition labeled Beginning, the signal appeared immediately after the action and lasted 0.5 s, whereas in the condition End, the signal appeared 5.5 s after the action. In the control No-Signal condition, there was no signal between the action and the outcome. Participants experienced a 6-s delay between action and outcome in all conditions. It took roughly 15 min to complete the experiment. The average experienced contingency in the PR conditions was 0.62.

### Results

#### Instrumental performance

Figure 3b shows a higher rate of responding in the Beginning compared to No-Signal and End conditions, suggesting a facilitatory effect of the signal only when experienced close to the action. A 2 (Contingency) × 3 (Signal) within-subjects ANOVA revealed a significant effect of Signal *F*(2,160) = 8.75, *p* <.001, η^*2*^_*p*_ = .10, 95% CI [.02, .19]. Further comparisons revealed differences between No-Signal and Beginning conditions, *F*(1,80) = 16.79, *p* < .001, η^*2*^_*p*_ = .17, [.05, .32], suggesting potentiation by the presence of the signal. However, there was no difference between No-Signal and End conditions, *F*(1,80) = 0.25, *p* = .620, suggesting no competition. Finally, there was a difference when comparing Beginning versus End conditions, *F*(1,80) = 13.71, *p* < .001, η^*2*^_*p*_ = .15, [.03, .28], revealing that the signal’s temporal position played a critical role.

#### Causal judgements

Figure 3c suggests an analogous effect of the presence of the signal in causal attribution. The same within-subjects ANOVA revealed the critical main effect of Signal *F*(2,160) = 14.46, *p* < .001, η^*2*^_*p*_ = .15, 95% CI [.04, .29]. Planned comparisons revealed a significant difference between No-Signal and Beginning, *F*(1,80) = 27.01, *p* < .001, η^*2*^_*p*_ = .25, [.10, .39], but not between No-Signal and End, *F*(1,80) = 3.35, *p* = .071. The response of the Beginning conditions was higher relative to the End, *F*(1,80) = 11.84, *p* = .001, η^*2*^_*p*_ = .13, [.02, .27].

The signal facilitated Action-Outcome learning but only when it was placed close to the action. Actually, the effect sizes reported here were numerically larger compared to the facilitation effects of Experiment 1. Moving the signal closer to the action boosted facilitation. However, when the signal was contiguous to the outcome, facilitation did not occur and nor did overshadowing. Hence, a strong Signal-Outcome contiguity per se was not sufficient to produce overshadowing.

## Experiment 3

Experiment 3 further manipulated a variable, salience of the signal, which was expected to promote competition by increasing elemental encoding. A compound formed by two stimuli that differ in salience has a profound impact on the magnitude of overshadowing (Mackintosh, 1976). Similarly, unequal saliences impair discriminations that require a configural solution, such as the biconditional discrimination (Byrom & Murphy, 2019). Thus, an increase in the salience of the signal should reduce configural processing and increase the magnitude of overshadowing. In Experiment 3, we used strong contiguity conditions (2-s delay), and manipulated the physical intensity of the signal, anticipating stronger overshadowing with a high-intensity signal.

### Method

#### Participants

Eighty-two (14 males) undergraduate students with an average age of 19.58 years (18–30 range) from the University of Leicester were recruited through the SONA system and participated in exchange for course credits.

#### Procedure and design

**Fig. 4:**
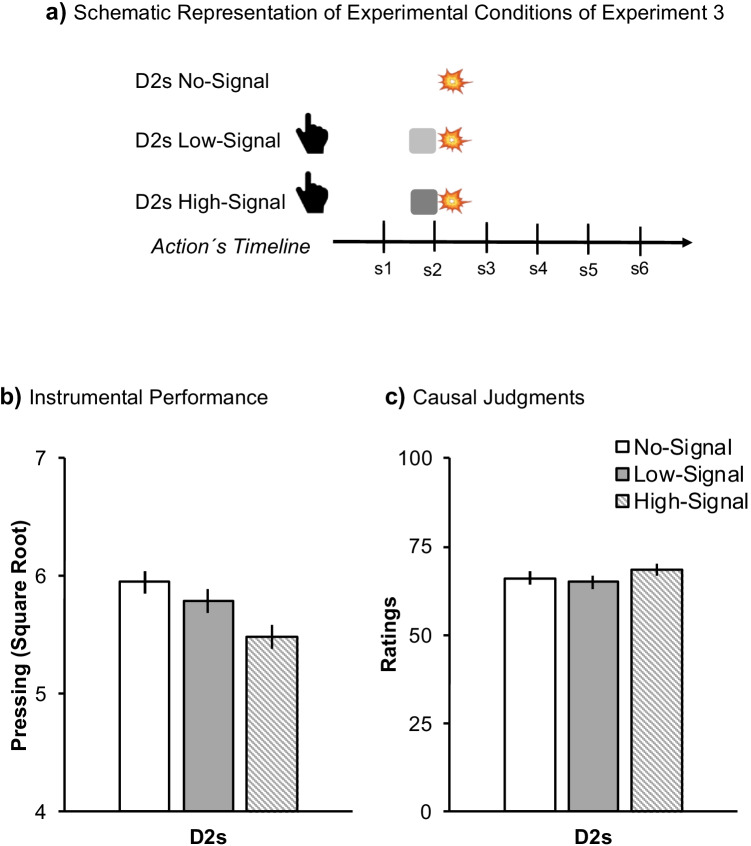
Design and results of Experiment 3. Panel (**a**) depicts the schematic timeline of each experimental condition for Experiment 3. D2s means that the outcome was delayed for 2 s. The hand symbolizes the action, the light-grey square represents the Low-Signal condition (similar to Experiments 1 and 2), and the dark-grey represents the High-Signal condition. Panel (**b**) represents the mean square root of the number of presses. Panel (**c**) depicts the mean of causal judgments. Error bars are SEM applying the within-subjects correction suggested by O’Brien and Cousineau (2014)

Figure 4a summarizes the experimental conditions of Experiment 3. The procedure was similar to Experiment 2, except that the current experiment involved six different experimental conditions resulting in a 2 × 3 within-subjects factorial design: 2 (Contingency: FR1 vs. Partial) × 3 (Signal: No-Signal, Low-signal, High-Signal). The factor Signal contained three different levels. In the condition Low-Signal, the sky changed to a powder grey color (similar to Experiments 1 and 2, Fig. 1c), while in the High-Signal condition the sky changed to a bright white color (Fig. 1d). The signal was presented during the final 0.5 s of the 2-s delay between Action and Outcome. In the control condition (No-Signal), there was no Signal presented between the Action and the Outcome. All conditions employed a 2-s delay. It took roughly 15 min to complete the experiment. Note that the average experienced contingency in the Partial conditions was 0.65.

### Results

#### Instrumental performance

Figure 4b shows a graded effect of Signal salience, with the lowest pressing in the High-Signal condition. A 2 (Contingency) × 3 (Signal) within-subjects ANOVA revealed the expected main effect of Signal *F*(2,162) = 5.55, *p* = .005, η^*2*^_*p*_= .06, 95% CI [.01, .14]. Further pairwise comparisons revealed differences between No-Signal and High-Signal conditions, *F*(1,81) = 10.62, *p* = .002, η^*2*^_*p*_ = .12, [.02, .25], suggesting overshadowing. However, there was no difference comparing No-Signal versus Low-Signal *F*(1,81) = 1.36, *p* = .246. Finally, the High-Signal condition yielded a lower number of presses than the Low-Signal, *F*(1,81) = 4.39, *p* = .039, η^*2*^_*p*_= .05, [.00, .17]. Overall, the No-Signal condition yielded a higher rate of pressing than both signal conditions collapsed, *F*(1,81) = 6.86, *p* = .010, η^*2*^_*p*_ = .08, [.01, .20], suggesting an overall decrement when there was a signal.

#### Causal judgements

Figure 4c suggests that the judgments were immune to the presence or salience of the signal. A similar within-subjects ANOVA did not revealed the main effect of Signal *F*(2,162) = 0.91, *p* = .405.

## Discussion

Across three experiments, an intervening signal led to competition or facilitation of Action-Outcome learning as a function of temporal contiguity. With strong Action-Outcome contiguity the signal overshadowed the action, reducing participants’ performance (Experiments 1 and 3). Moreover, a more salient signal yielded stronger overshadowing (Experiment 3). However, under conditions of weak contiguity the very same signal facilitated both performance and causal attribution, but only when the signal was placed relatively close to the action (Experiments 1 and 2). To our knowledge, this is the first evidence in human participants showing that signals can both compete with and facilitate actions, depending on Action-Outcome temporal contiguity (Schachtman et al., 1987, in pigeons).

These results suggest that cue-interaction phenomena depend on temporal contiguity (Urcelay, 2017). We have recently proposed (Herrera et al., 2022) that the dependence of competition phenomena on contiguity can be accommodated by a modification of standard configural theory (Pearce, 1987). Pearce’s original model explains overshadowing as an instance of generalization decrement, which is the decline in responding caused by a change from training (e.g., Action-Signal) to test (e.g., Action). Pearce´s model computes the similarity between the trained compound and the test stimulus, and this depends on the relative proportion of unique elements and common elements that the stimuli share. Our modification advocates that the way to compute similarity largely depends on contiguity. With strong contiguity, unique elements are well remembered, and hence predicts strong generalization decrement (more unique than common elements) – that is overshadowing. However, a delayed outcome might allow some time for unique elements to decay, increasing control by common elements and resulting in broader generalization from training to test. Although the notion that time passage broadens generalization gradients is by no means new (Pavlov, 1927; Riccio et al., 1994; see Buriticá & Alcalá, 2019, for interval timing), it allows for an integration of contiguity at a theoretical level to account for its critical effects on competition and facilitation. Thus, in Experiments 1 and 2, the conditions of weak contiguity caused the representation of Action-Signal to become configured, and this facilitated instrumental performance by increasing generalization from the Action-Signal compound to the Action. Indeed, in Experiment 2 the signal only facilitated performance when experienced close to the action, supporting this notion of configural processing. Finally, Experiment 3 showed that a more salient signal produced a larger overshadowing, in line with the notion that increases in elemental processing result in larger generalization decrement.

The majority of trial-based associative learning models were developed assuming strong temporal contiguity between events (see Boakes & Costa, 2014). Thus, these models have largely focused on competition, because competition is the most likely outcome with strong contiguity. Although they invariably predict lower performance with delayed outcomes, they make no clear predictions about the magnitude of overshadowing as a function of contiguity. However, temporal difference reinforcement learning models (TD), characterized by moment-to-moment updates of learning, can predict less overshadowing as a function of outcome timing (Ludvig et al., 2012). When CSs in a compound differ in length, if CS1 starts earlier than CS2 (but both co-terminate before the outcome is presented; i.e., strong contiguity), the CS1–CS2 interval determines the magnitude of overshadowing: shorter CS1–CS2 intervals result in strong overshadowing, but longer intervals attenuate overshadowing. Assuming that Action plays the role of CS1 (Signal = CS2), it is possible for TD models to predict attenuated overshadowing. That is, in our conditions where the signal preceded the outcome, we observed overshadowing with short (but not long) Action-Outcome intervals, in line with the prediction of TD models. However, it is less clear how these models can handle the potentiation of actions by signals that we observed in Experiments 1 and 2. Overall, what is clear is that outcome timing determines the size of the overshadowing effect.

We observed somewhat convergent evidence from the two measures we used. However, the intervening signal seems to impact behavioral performance more than causal attribution, suggesting a dissociation between these measures (Pérez & Soto, 2020). Delayed outcomes in human participants have revealed that signals tend to facilitate causal attribution, with less influence on instrumental performance (Reed, 1992, 1999; Shanks, 1989). We are only aware of two reports showing that a signal competes with Action-Outcome learning (Hammerl, 1993; Lovibond et al., 2013). The discrepancies observed can be accounted for by two factors. First, our signal plays an ambiguous role (it might indicate that either the participant, or the participant’s comrades, are shooting). Previous research suggests that expectations about outcome timing attenuate the deleterious effect of delayed outcomes (Buehner & May, 2004). As a consequence, a less ambiguous signal might have promoted competition in causal attribution. Second, the ratio schedule of reinforcement used in this series may have promoted facilitation, in contrast to interval schedules of reinforcement, which favor competition (Hammerl, 1993; Reed et al., 1988). This may have particularly affected causal judgments (preventing competition), because ratio-schedules promote goal-directed behaviors (Dickinson et al., 1983). Future studies should extend the present results using different instructions and interval reinforcement schedules.

Overall, the present results challenge the widely accepted notion of competition when there are multiple predictors of an outcome, and advance our understanding of the interactions between environmental signals and actions in the pursuit of delayed goals.

## Supplementary Information

ESM 1 (DOCX 40 kb)

## Footnotes

1 The Action-Signal-Outcome arrangement creates different contiguity relationships. We are mainly interested in the contiguity between Action-Outcome. However, other relationship may be also playing an important role as a function of contiguity between Action-Signal and Signal-Outcome. Although all potential associations may influence Action-Outcome learning, our expectations are mainly based on the contiguity between Action-Outcome. However, as explored in Experiment 2 the Action-Signal contiguity also plays a critical role determining facilitation.

2 The main effect of Replication was significant *F*(1,79) = 4.63, *p* = .034, η^*2*^_*p*_ = .05, 95% CI [.00, .17], revealing that instrumental performance was overall higher during Replication 2 (*M* = 5.68, *SD* = 1.67) compared with Replication 1 (*M* = 4.90, *SD* = 1.43). This was likely due to the use of the extra sentence “*“However, it is in your best interest to conserve your ammo and not fire constantly”* in the instructions in Replication 1. However, replication did not interact with the critical Delay x Signal interaction.
