# Supplementary material for: Temporal contiguity determines overshadowing and potentiation of human Action-Outcome performance
Source: Psychon Bull Rev. 2022 Aug 11;30(1):350–61. doi: 10.3758/s13423-022-02155-4 (PMC9371369; doi:10.3758/s13423-022-02155-4)
Supplement: Supplementary file 1 — (DOCX 40 kb) [file 13423_2022_2155_MOESM1_ESM.docx]

**Temporal contiguity determines overshadowing and potentiation of human Action-Outcome performance**

José A. Alcalá, Richard D. Kirkden, Jess Bray, José Prados, & Gonzalo P. Urcelay

**1. Supplementary Analyses**

Analyses of the Triple interaction Contingency x Trace x Signal mentioned in Footnote 2 in Experiment 1 for causal judgments.

The triple interaction Contingency x Trace x Signal was significant, *F*(1, 79) = 4.86, *p* = .030, η*^2^_p_* = .06, 95% CI [.00, .18]. Further analyses of this triple interaction showed that the Trace x Signal interaction was significant only in the deterministic contingency, *F*(1, 80) = 11.60, *p* = .001, η*^2^_p_* = .13, 95% CI [.02, .26], but not in the probabilistic contingency conditions, *F*(1, 80) = 0.97, *p* = .327. However, further pairwise comparisons, revealed that these were similar. In the case of FR1 contingency, (see figure 1) there was no effect of signal in the 2s trace conditions, *F(*1,80*) =* 1.42*, p =* .233, η*^2^_p_* = .02, 95% CI [.00, .11] but there was in the 6s conditions *F*(1, 80) = 11.60, *p* = .001, η*^2^_p_* = .13, 95% CI [.02, .26] with higher ratings in the presence of the signal than in its absence.

**Figure 1**

Note: *Mean of causal judgments for Experiment 1 with FR1 contingency in each level of delay. D2s refers to the condition in which the outcome was delayed 2s and D6s refers to 6s delay. Error bars are SEM applying the within-subjects correction suggested by O’Brien & Cousineau (2014)*

In the Partial Reinforcement conditions (see Figure 2), there was no effect of signal in 2s trace conditions, *F*(1,80) = 1.33, *p* = .252; but there was an effect in the 6s trace conditions with higher responding in the presence of the signal compared to the absence of the signal (, *F*(1, 80) = 5.83, *p* = .018, η*^2^_p_* = .07, 95% CI [.001, 19].

**Figure 2**

Note. *Mean of causal judgments for Experiment 1 with Partial Reinforcement contingency in each level of delay. D2s refers to the condition in which the outcome was delayed 2s and D6s refers to 6s delay. Error bars are SEM applying the within-subjects correction suggested by O’Brien & Cousineau (2014)*

**2. Descriptive Statistics**

**Experiment 1**

|  | Instrumental Performance | | Causal Judgments | |
| --- | --- | --- | --- | --- |
| Condition | Mean | SD | Mean | SD |
| D2 No-Signal | 5.72 | 1.88 | 65.57 | 24.66 |
| D2 Signal | 5.41 | 1.71 | 65.33 | 25.04 |
| D6 No-Signal | 5.10 | 1.71 | 44.92 | 27.45 |
| D6 Signal | 5.36 | 1.76 | 56.62 | 27.62 |

*Mean and Standard Deviation for each experimental conditions collapsing data across replication and contingencies for Instrumental Performance and Causal Judgments.*

**Experiment 2**

|  | Instrumental Performance | | Causal Judgments | |
| --- | --- | --- | --- | --- |
| Condition | Mean | SD | Mean | SD |
| D6 No-Signal | 5.12 | 2.01 | 30.98 | 25.95 |
| D6 Beginning | 5.74 | 1.68 | 48.06 | 26.34 |
| D6 End | 5.01 | 1.90 | 36.66 | 23.10 |

*Mean and Standard Deviation for each experimental conditions collapsing data across contingencies for Instrumental Performance and Causal Judgments.*

**Experiment 3**

|  | Instrumental Performance | | Causal Judgments | |
| --- | --- | --- | --- | --- |
| Condition | Mean | SD | Mean | SD |
| D2 No-Signal | 5.95 | 1.83 | 66.10 | 20.50 |
| D2 Beginning | 5.49 | 1.81 | 68.51 | 22.95 |
| D2 End | 5.79 | 1.76 | 65.05 | 22.51 |

*Mean and Standard Deviation for each experimental conditions collapsing data across contingencies for Instrumental Performance and Causal Judgments.*
